# Epidemiology and Control of Legionellosis, Singapore

**DOI:** 10.3201/eid1707.101509

**Published:** 2011-07

**Authors:** Meng Chon Lam, Li Wei Ang, Ai Ling Tan, Lyn James, Kee Tai Goh

**Affiliations:** Author affiliations: Ministry of Health, Singapore (M.C. Lam, L.W. Ang, L. James, K.T. Goh);; Singapore General Hospital, Singapore (A.L.Tan)

**Keywords:** legionellosis, Legionella, epidemiology, bacteria, cooling towers, water fountains, artificial water systems, legislation, Singapore, research

## Abstract

To determine trends and clinical and epidemiologic features of legionellosis in Singapore, we studied cases reported during 2000–2009. During this period, 238 indigenous and 33 imported cases of legionellosis were reported. Cases were reported individually and sporadically throughout each year. Although the annual incidence of indigenous cases had decreased from 0.46 cases per 100,000 population in 2003 to 0.16 cases per 100,000 in 2009, the proportion of imported cases increased correspondingly from 6.2% during 2000–2004 to 27.3% during 2005–2009 (p<0.0005). The prevalence of *Legionella* bacteria in cooling towers and water fountains was stable (range 12.1%–15.3%) during 2004–August 2008.

Legionellosis (Legionnaires’ disease and Pontiac fever) is an environment-related, acute respiratory infection caused by gram-negative, rod-shaped bacteria of the genus *Legionella*. Pontiac fever is a self-limiting influenza-like syndrome; Legionnaires’ disease is more severe, has pneumonia as the predominant clinical finding, and is a potentially fatal illness. Currently, there are 52 *Legionella* species (*1*) and 70 serogroups (*2*). Of these species, 25 species are known to cause human disease (*1*). Most human infections are caused by *Legionella pneumophila* (*3*), and the predominant serogroup is serogroup 1 (*4*). Other species, which together with *L*. *pneumophila*, account for most human infections include *L*. *longbeachae* and *L*. *micdadei*. The mode of transmission of legionellosis is believed to be by inhalation of aerosols (*5*). Other possible modes of transmission such as aspiration of contaminated potable water have also been widely discussed (*6**–**8*).

Legionellosis was first identified in 1976 during an outbreak of severe pneumonia among delegates to the 1976 American Legion Convention in Philadelphia (*9*). Since then, several outbreaks linked to a variety of aerosol-producing devices, such as cooling towers (*10**–**13*), whirpool spas (*14*), decorative fountains (*15*), mist machines (*16**,**17*) and industrial air scrubbers (*18*), have been reported. The largest outbreak of the disease (449 confirmed cases) was attributed to cooling towers in a city hospital in Murcia, Spain, in July 2001 (*10*).

Risk factors for legionellosis include cigarette smoking, chronic lung disease, and immunosuppression (especially that caused by corticosteroid therapy and organ transplantation) (*8*). Environmental factors such as high humidity and increased rainfall also increase the risk for legionellosis (*19*).

In Singapore, a densely populated, tropical city-state with many high-rise commercial, office, and residential air-conditioned buildings, Legionnaires’ disease was recognized as a potential public health threat because environmental surveys showed that cooling towers were heavily colonized by *Legionella* spp. bacteria. A study conducted in 1987 showed that *Legionella* spp. were present in 7 (46.7%) of 15 cooling towers sampled (*20*). Because air-conditioning systems are operated most of the year, a large heat load is imposed on water cooling systems, thus facilitating an increased rate of colonization and multiplication of *Legionella* spp (*21*). Furthermore, the viability of *Legionella* spp. in contaminated aerosols is increased by high relative humidity (*21*).

*Legionella* spp. pneumonia accounts for 2%–7% of community-acquired pneumonia among hospitalized patients in Singapore (*22*). Legionnaires’ disease was made a notifiable infectious disease in Singapore in 1985 and legally notifiable in 2000. A code of practice for prevention and control of *Legionella* spp. bacteria in cooling towers for building owners and water treatment contractors was published in 1992 and revised in 1994 and 1998. Subsequently, Environmental Public Health (Cooling Towers and Water Fountains) Regulations were enacted and implemented nationwide in 2001 (*23*).

We studied trends and clinical and epidemiologic features of legionellosis in Singapore during 2000–2009. We also reviewed the prevalence of *Legionella* spp. bacteria in cooling towers and water fountains during the same period to determine whether any relationship existed between disease incidence and prevalence of *Legionella* spp. bacteria in these artificial water systems during the past decade.

## Materials and Methods

### Case Surveillance

The Ministry of Health (MOH) is responsible for surveillance of legionellosis in Singapore. The Infectious Diseases Act requires all registered medical practitioners and directors of clinical laboratories to notify the MOH of all cases of legionellosis by fax or electronically through a dedicated website by using a standard notification form within 24 hours after diagnosis. Clinical and laboratory criteria for notification are based on the guidelines published by the MOH (*24*). Pontiac fever is defined as a self-limiting influenza-like illness with malaise, myalgia, fever, chills, and headache without pneumonia, and Legionnaires’ disease is defined as a clinical syndrome and symptoms with fever, myalgia, headache, cough, chest pain, anorexia, nonbloody diarrhea, encephalopathy, or change in sensorium, with pneumonia as the predominant clinical finding. A confirmed case of Legionnaires’ disease or Pontiac fever is defined as a clinically compatible disease with a 4-fold increase in *Legionella* spp. antibody titer in paired serum samples, or presence of *Legionella* spp. antigen in urine (BinaxNOW *Legionella* immunochromatographic test; Binax Inc., Portland, OR, USA) or positive immunofluorescence or isolation of *Legionella* spp. from respiratory specimens. If clinical diagnosis was based on a positive *Legionella* spp. antibody titer >1,024 in a serum sample, the case was classified as presumptive.

Each reported case was investigated thoroughly by a trained public health officer by using a standard questionnaire. Investigations included interviews with the case-patient or family members and a review of clinical and laboratory records. Relevant epidemiologic and clinical data obtained included age, sex, ethnic group, occupation, residential status, residential and work place addresses, clinical features, onset of illness, recent history of travel and hospitalization before onset of illness, concurrent conditions, and clinical outcomes. Investigators paid special attention to identify any clustering of cases by person, place, and time. A cluster was defined as a locality where >2 cases occurred within 6 months of each other and with the same residential or workplace addresses within 500 m of each other. If the case-patient had a recent travel history outside Singapore 10 days before the onset of illness, the case was considered imported. We analyzed clinical and epidemiologic data maintained by the Communicable Diseases Division, MOH, during 2000–2009.

### Environmental Surveillance

The National Environment Agency, Ministry of Environment and Water Resources, is responsible for environmental surveillance and control of *Legionella* spp. bacteria in cooling towers, water fountains, and other artificial water systems in Singapore. It works closely with the MOH to prevent outbreaks of legionellosis.

Under legislation enacted in Singapore, water samples obtained from cooling towers and water fountains are submitted to any of the 7 Singapore Accreditation Council-Singapore Laboratory Accreditation Scheme laboratories, which have been accredited to perform testing for *Legionella* spp. in Singapore. Water samples of ≈500 mL were obtained from the pond of the cooling tower or fountain by using a pump that had been disinfected with sodium hydrochlorite and neutralized with sodium thiosulfate. Testing of *Legionella* spp. is based on international laboratory standards such as the BS 6068–4.12:1998, AS/NZS 3896:1998, and ISO 11731:1998 (*25*). The limit of detection for the most commonly used standard (BS 6068–4.12:1998) in the Food and Water Microbiology Laboratory at the Singapore General Hospital (SGH) was 100 CFU/L.

Samples were first concentrated 100-fold by using membrane filtration, heated at 50°C for 30 min, and treated with acid by using HCl–KCl buffer, pH 2.2, for 5 min. Concentrated samples were then plated on charcoal yeast extract agar supplemented with cysteine, ferric ions, and antimicrobial drugs (Oxoid Ltd., Basingstoke, UK). These culture plates were incubated at 36°C ± 1°C and examined at regular intervals for a maximum of 10 days. Colonies suspected of being *Legionella* spp. were then investigated by using biochemical and serologic tests. Speciation or serogrouping of *Legionella* spp. isolates was done by using a direct fluorescent antibody test and latex agglutination or slide agglutination. For every sample, the culture plate showing the maximum number of confirmed *Legionella* spp. colonies was used to estimate the number of CFUs of *Legionella* spp. in the original water sample.

The Food and Water Microbiology Laboratory at SGH was the reference laboratory for the testing of *Legionella* spp. bacteria in Singapore. However, as of September 2008, it ceased testing of *Legionella* spp. bacteria in water samples. We analyzed data maintained by the Food and Water Microbiology Laboratory, SGH, because it was the only public sector laboratory with reliable records.

### Data Analysis

The estimated mid-year population of the corresponding years obtained from the Department of Statistics, Singapore, was used to calculate incidence rates. Statistical analyses were performed by using SPSS software version 17.0 (SPSS Inc., Chicago, IL, USA). Linear patterns in age distribution of legionellosis case-patients over time were assessed by using the χ^2^ test for trend. Association between medical conditions and whether the case-patient survived or died was examined by using the Fisher exact test. In all data analyses, a p value <0.05 was considered significant.

## Results

During 2000–2009, a total of 238 indigenous and 33 imported cases of legionellosis were reported in Singapore. An additional 4 cases reported among tourists and 48 among foreigners who sought medical treatment in Singapore were excluded. Of the 271 reported cases included in the study, 195 were classified as Legionnaires’ disease (72 confirmed and 123 presumptive) and 76 as Pontiac fever (9 confirmed and 67 presumptive) (Table 1). Although none of the cases were diagnosed by immunofluorescent assay or culture of respiratory specimens, the percentage of cases diagnosed by using the *Legionella* urinary antigen test increased from 10.8% in 2000 to 19.0% in 2005 and 45.5% in 2009.

**Table 1:** Classification of reported indigenous and imported legionellosis cases, by year, Singapore, 2000–2009*

Classification	2000	2001	2002	2003	2004	2005	2006	2007	2008	2009	Total
Confirmed											
Pontiac fever	0	4	1	3	1	0	0	0	0	0	9
Legionnaires’ disease	3	15	12	16	4	5	1	0	5	11	72
Presumptive											
Pontiac fever	7	12	9	16	4	0	0	4	8	7	67
Legionnaires’ disease	47	15	15	6	4	13	12	8	2	1	123
Total	57	46	37	41	13	18	13	12	15	19	271

*Four tourists and 48 foreigners seeking medical treatment in Singapore were excluded.

The highest mean annual age-specific incidence per 100,000 population was in persons 75–84 years of age; the male:female ratio was 1.4:1 (Table 2). The proportion of case-patients >55 years of age increased significantly from 44.4% in 2004 to 76.1% in 2009 (p<0.0005). Among the 3 major ethnic groups, Indians had the highest mean annual incidence rates (0.83 cases/100,000 population), followed by Chinese (0.70 cases/100,000 population) and Malays (0.55 cases/100,000 population). Retirees (32.8%) constituted most of the reported case-patients, followed by professionals, self-employed persons, and managers (17.7%); and housewives (17.2%). Cases occurred singly and sporadically throughout the year, and no cluster was detected. None of the case-patients had a recent history of hospitalization within 2 weeks before onset of illness.

**Table 2:** Age and sex distribution, age-specific incidence, number of deaths, and CFRs among 271 case-patients with indigenous or imported legionellosis, Singapore, 2000–2009*

Age group, y	M	F	Total	Mean incidence†	No. deaths	CFR, %
0–4	1	0	1	0.05	0	0
5–14	1	0	1	0.02	0	0
15–24	7	1	8	0.13	0	0
25–34	11	6	17	0.20	0	0
35–44	19	8	27	0.36	1	3.7
45–54	24	12	36	0.61	0	0
55–64	27	18	45	1.38	2	4.4
65–74	37	33	70	3.73	1	1.4
75–84	28	25	53	6.66	2	3.8
>85	3	10	13	5.83	0	0
Total	158	113	271	0.65	6	2.2

*Four tourists and 48 foreigners seeking medical treatment in Singapore were excluded. CFR, case-fatality rate. †Per 100,000 population. Based on estimated mid-year population of 2004.

The annual incidence rate of indigenous cases (confirmed and presumptive) decreased markedly from 1.37/100,000 population in 2000 to 0.28/100,000 population in 2009 (p = 0.001) (Figure) and correspondingly increased in imported cases from 6.2% during 2000–2004 to 27.3% during 2005–2009 (p<0.0005) (Figure). The countries of origin of these imported cases were mainly in Asia. The annual incidence rate for conformed indigenous cases also decreased from 0.46 per 100,000 population in 2003 to 0.16 per 100,000 population in 2009. The overall case-fatality rate (CFR) was 2.2% for confirmed and presumptive cases and 3.7% for confirmed cases.

**Figure:**
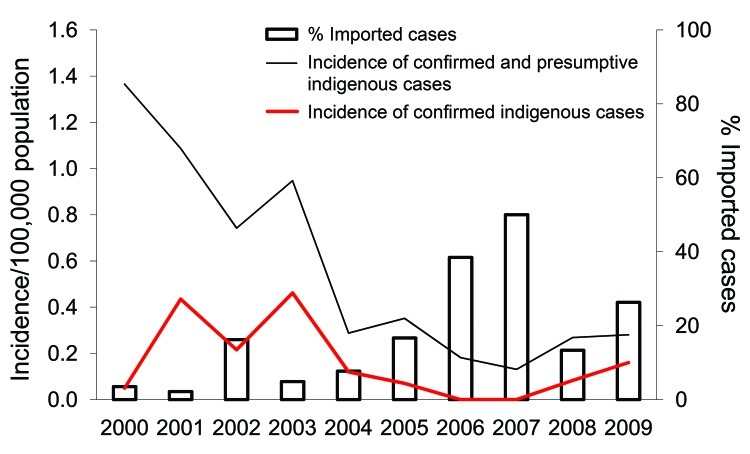
Incidence rate (per 100,000 population) of indigenous legionellosis cases and proportion (%) of imported cases, Singapore, 2000–2009.

The main clinical features of reported cases were cough (77.9%), fever (72.7%), shortness of breath (32.5%), chest pain or discomfort (13.3%) and nausea or vomiting (11.8%) (Table 3). Most (52.8%) case-patients had concurrent conditions such as hypertension (31.5%), diabetes mellitus (23.8%), ischemic heart disease (16.1%), renal failure (11.9%), and asthma (11.2%). Six deaths were reported; 3 in 2000, 1 in 2001, 1 in 2002, and 1 in 2009. All but 1 death occurred in persons >55 years of age, and all persons who died had concurrent conditions of the cardiovascular system. The CFR for persons with concurrent conditions (6/143, 4.2%) was significantly higher than that for persons without these conditions (0/128, 0%) (p = 0.031).

**Table 3:** Signs and symptoms among 271 case-patients with indigenous or imported legionellosis, Singapore, 2000–2009*


Fever (with/without chills and rigors)	197 (72.7)
Respiratory	
Cough (productive or nonproductive)	211 (77.9)
Shortness of breath	88 (32.5)
Chest pain and discomfort	36 (13.3)
Runny nose	4 (1.5)
Rhinorrhea	1 (0.4)
Bronchitis	2 (0.7)
Sore throat	7 (2.6)
Gastrointestinal	
Nausea/vomiting	32 (11.8)
Abdominal/epigastric pain	13 (4.8)
Diarrhea	8 (3.0)
Neurologic	
Drowiness/giddiness	13 (4.8)
Headache	9 (3.3)
Other signs or symptoms	
Chills	31 (11.4)
Generalized weakness	10 (3.7)
Myalgia	5 (1.9)
Lethargy	8 (3.0)
Loss of appetite	15 (5.5)
Others	22 (8.2)

*Four tourists and 48 foreigners seeking medical treatment in Singapore were excluded. †Case-patients may have >1 sign or symptom.

### Surveillance of *Legionella* Bacteria in the Environment

Based on available data, 18,164 water samples from cooling towers and 1,277 water samples from water fountains were tested at the Food and Water Microbiology Laboratory, SGH, during 2000–2008. All samples were random samples routinely collected by water treatment contractors, building managers, and environmental health officers from the former Quarantine and Epidemiology Department, Ministry of the Environment and Water Resources. Data for 2000–2002 was obtained from annual reports of the Quarantine and Epidemiology Department and was a subset of the total number of samples tested in this laboratory. Data for 2003 were unavailable because this department kept its records for only 5 years before discarding them. The mean positivity rates for *Legionella* spp. bacteria were 15.6% for cooling tower samples and 12.4% for water fountain samples. Isolation rates for *Legionella* spp. decreased significantly in cooling towers and water fountains from an average of 58.4% during 2000–2002 to an average of 13.7% during 2004–2008 (p<0.0005) (Table 4).

**Table 4:** Isolation rate for *Legionella* bacteria in environmental samples obtained from cooling towers and fountains, Singapore, 2000–August 2008*

Characteristic	No. positive/no. tested (%)							
	2000†	2001†	2002†	2004	2005	2006	2007	2008‡
Isolation rate	114/193 (59.1)	220/323 (68.1)	140/291 (48.1)	883/7,284 (12.1)	635/4,160 (15.3)	448/3,073 (14.6)	385/2,711 (14.2)	172/1,406 (12.2)

*Data were unavailable for 2003. †Data were incomplete. ‡January–August only.

## Discussion

The incidence of legionellosis in Singapore has decreased during the past decade. Its incidence for indigenous cases in 2009 was 0.28 cases per 100,000 population (0.16/100,000 population for confirmed cases only), which was much lower than that in Europe (1.18/100,000 in 2008) (*26*) and Hong Kong (0.53/100,000 in 2009) (*27*). The percentage of imported cases of legionellosis has also increased over the past 5 years from 6.2% during 2000–2004 to 27.3% during 2005–2009 (p<0.0005). Imported legionellosis is often related to overnight stays in public accommodations (*28*). The number of imported cases is expected to increase further because of improved reporting and surveillance and increasing regional and international travel. A surveillance information exchange system similar to the European Surveillance Scheme for Travel Associated Legionnaires’ Disease (*28*) should be established in the Asia–Pacific region so that member countries could detect possible clusters of imported legionellosis that would have been undetected by national surveillance systems in individual countries. Regional and international cooperation in the sharing of best practices would also be useful.

The epidemiologic and clinical features of legionellosis in Singapore are comparable to those in temperate countries (*2**,**27**,**29*). The incidence rate was highest for persons 75–84 years of age; there was a slight predominance in male patients and in patients with concurrent conditions. Retirees were more susceptible to legionellosis than were persons with other occupations, which also reflected higher disease incidence for persons >65 years of age. During 2000–2009, the overall CFR was 2.2% for confirmed and presumptive cases and 3.7% for confirmed cases, which was lower than that in Europe (6.5% in 2008) (*26*). The CFR was higher for persons with concurrent conditions. The CFR was recently reduced in comparison with the rate (14.7%) during 1986–1996 (*30*). This reduction could be attributed to wider use of *Legionella* urinary antigen tests in the diagnosis of Legionnaires’ disease, which led to faster diagnosis than with traditional serologic tests. The number of cases diagnosed by using the *Legionella* antigen test increased from 10.8% in 2000 to 19.0% in 2005 and 45.5% in 2009. A delay in appropriate antimicrobial drug therapy has been associated with an increased mortality rate (*31*).

Clinical practice guidelines of the Singapore MOH for use of antimicrobial drugs in adults, which have been in use since 2000, had recommended antimicrobial drugs active against *Legionella* spp., specifically macrolides (erythromycin, clarithromycin, and azithromycin) or fluoquinolones (levofloxacin) (*32**,**33*) as first-line empirical treatment for community-acquired pneumonia in persons because of their risk category. However, laboratory testing for Legionnaires’ disease is needed to ensure that patients are adequately treated for this disease. Thus, results of *Legionella* urinary antigen tests would assist clinicians in formulating antimicrobial drug therapy and result in a lower CFR.

During the same period, prevalence of *Legionella* spp. bacteria decreased in cooling towers and water fountains in Singapore. This finding could have been caused by the Environmental Public Health (Cooling Towers and Water Fountains) Regulations, which were enacted and implemented nationwide in 2001. This legislation specifies the frequency of inspection, maintenance, and testing of water for *Legionella* spp. bacteria. Cooling towers or water fountain should be thoroughly cleaned and disinfected at least once every 6 months and inspected at least once a week for any physical defect, general cleanliness, organic fouling, and physical debris. Furthermore, water samples should be sampled and tested by a government laboratory or any Singapore Accreditation Council–Singapore Laboratory Accreditation Scheme–accredited laboratories at least once a month to determine standard plate counts and at least once every 3 months to detect *Legionella* spp.

The owner or occupier of these water facilities is required to keep records of any remedial or maintenance work, inspection, or test conducted and to make available such records for inspection by any public health officer from the National Environment Agency. If cooling towers or water fountains were found by the Director-General of Public Health to endanger health of any person, the health authority could require the owner or occupier to stop using the cooling tower or water fountain and to cordon off the immediate vicinity. For first offense, the penalty is a fine not exceeding 5,000 Singapore dollars. For a second offense and subsequent offenses, the penalty is a fine not exceeding 10,000 Singapore dollars (*23*).

It is tempting to attribute the decreasing disease trend to introduction of legislation to prevent and control *Legionella* spp. bacteria in cooling towers and water fountains. However, there are several limitations that should be considered before any conclusion could be made for this relationship. In addition, other factors could have also contributed to the decrease of indigenous cases of legionellosis in Singapore.

First, no epidemiologic evidence exists to link reported cases of legionellosis to environmental isolates of *Legionella* spp. because these bacteria has never been isolated from infected patients in Singapore since the disease became notifiable in 1985. Although isolation of *Legionella* spp. from respiratory specimens is the standard in the diagnosis of legionellosis, this laboratory method is not the method of choice for many clinicians because there are other more convenient methods for testing.

Second, *Legionella* spp. bacteria are ubiquitous in the environment. Their prevalence in other artificial water systems was 31.8% in spa establishments (*34*). In another study conducted during 1998–2002, *Legionella* spp. bacteria were found in 16.2% of mist fans and 23.7% of water taps and shower heads (*35*). However, these bacteria were not detected in the municipal potable water system because the surveillance program of the Public Utilities Board (National Water Agency) started in 2003.

A major contributing factor that has possibly resulted in the decrease of *Legionella* spp. colonization in the potable water system was replacing chlorination with chloramination in disinfecting the potable water system since 2005. In 2008–2009, the Public Utilities Board (National Water Agency) further intensified its monitoring program by obtaining >130 samples quarterly from hospitals, medical centers, dead ends of the distribution system, and water taps but no *Legionella* spp. bacteria were detected. The results were again negative in a special study to detect *Legionella* spp. bacteria in the biofilm of the municipal potable water distribution system. The findings were reviewed by an external audit panel comprising local and overseas experts. Monochloramine disinfection of municipal water supplies is associated with decreased risk for Legionnaires’ disease (*36**,**37*). Thus, the use of chloramine since 2005 could have also led to the decreasing incidence of indigenous cases of legionellosis (confirmed and presumptive).

Third, the disease surveillance system favors identification of the more severe form of legionellosis, which represents a fraction of the actual extent of infections in the community. A seroepidemiologic survey of the general population in Singapore showed a prevalence of 10.3% for antibodies against *Legionella* spp. in persons <20 years of age and 21.9% in persons >20 years of age (*30*).

Fourth, the rapid immunochromatographic assay is more widely used for detection of *L*. *pneumophila* serogroup 1 antigen in urine specimens (*38*). This finding suggests that legionellosis caused by other species or serogroups would have been missed.

Fifth, most (70.1%) reported legionellosis cases were classified as presumptive, which would not constitute a definite case of Legionnaires’ disease in the United States and some other countries. A decreasing trend was also observed when we considered only confirmed indigenous cases. Although, the annual incidence rate for confirmed indigenous cases decreased from 0.46 per 100,000 population in 2003 to 0.16 per 100,000 population in 2009, this difference was not significant. This finding could have been caused by the smaller sample size (n = 67) of confirmed indigenous cases of legionellosis during 2000–2009.

Sixth, changes in clinical testing patterns may have resulted in the observed decrease in incidence rate. Unfortunately, except for the clinical laboratories at SGH, we did not have data from other hospitals and clinical laboratories where *Legionella* spp. testing was performed.

Seventh, the results for water samples obtained from cooling towers and water fountains and submitted to the Food and Water Microbiology Laboratory at SGH may not be representative of the situation in Singapore. Thus, results and conclusions derived from this study should be interpreted with caution. More research should be planned and conducted to evaluate the effectiveness of legislation in the control of legionellosis.

The absence of outbreaks of legionellosis, and the decrease in the incidence rate, CFR, and prevalence rate of *Legionella* spp. bacteria in the cooling towers and water fountains is reassuring. However, a high level of vigilance should continue in the maintenance and inspection of cooling towers and water fountains. Studies should be conducted to determine epidemiologic and molecular relationships between legionellosis cases and environmental *Legionella* isolates. At the same time, the prevalence of *Legionella* spp. bacteria in other aerosol-producing artificial water systems should be periodically assessed and appropriate preventive and control measures taken.
